# Increased doxorubicin uptake and toxicity in multicellular tumour spheroids treated with DC electrical fields

**DOI:** 10.1038/sj.bjc.6690487

**Published:** 1999-06

**Authors:** H Sauer, V Pütz, K Fischer, J Hescheler, M Wartenberg

**Affiliations:** Department of Neurophysiology, University of Cologne, Robert-Koch-Str. 39, Cologne, D-50931, Germany

**Keywords:** electrochemotherapy, electrical field, multicellular tumour spheroid, doxorubicin, lipid peroxidation, lipid diffusion

## Abstract

Electrochemotherapy (ECT) is a new approach to the treatment of tumours. In the present study, multicellular prostate tumour spheroids were treated with non-lethal direct current (DC) electrical fields, and uptake and toxicity of doxorubicin were investigated. An electrical field with a field strength of 500 Vm^−1^ applied for a duration of 90 s resulted in neither reversible nor irreversible membrane breakdown as revealed by fluid phase uptake studies of the membrane impermeant tracer Lucifer yellow. However, treated spheroids showed an increased uptake of doxorubicin and, consequently, an increased toxicity following electrical field exposure. The electrical field raised intracellular reactive oxygen species (ROS) as revealed using 2′,7′-dichlorofluorescein diacetate (H_2_DCFDA) as an indicator. ROS induced membrane lipid peroxidation since the lipid peroxidation end products malondialdehyde (MDA) and 4-hydroxy-2-(E)-nonenal (4-HNE) were detected after electrical field treatment. Moreover, lipid peroxidation decreased the lipid diffusion coefficient D from 4.2 × 10^−10^ cm^2^ s^−1^ to 2.7 × 10^−10^ cm^2^ s^−1^ in the control and treated sample, respectively, as revealed by fluorescence recovery after photobleaching (FRAP) experiments. The field effects could be mimicked by incubating spheroids with 100 nM hydrogen peroxide and were inhibited by the radical scavengers dehydroascorbate (DHA) and α-tocopherol (vitamin E), indicating that the increased uptake of doxorubicin after electrical field treatment is owing to lipid peroxidation and decreased membrane lipid mobility. Treatment of tumours with low intensity electrical fields may be useful to improve the cytotoxic capacity of anthracyclines. © 1999 Cancer Research Campaign


*British Journal of Cancer* (1999) **80**(8), 1204–1213
© 1999 Cancer Research Campaign
Article no. bjoc.1998.0487

### Increased doxorubicin uptake and toxicity in multicellular tumour spheroids treated with DC electrical fields

**H Sauer, V Pütz, K Fischer, J Hescheler and M Wartenberg**

Department of Neurophysiology, University of Cologne, Robert-Koch-Str. 39, D-50931 Cologne, Germany

**Summary** Electrochemotherapy (ECT) is a new approach to the treatment of tumours. In the present study, multicellular prostate tumour spheroids were treated with non-lethal direct current (DC) electrical fields, and uptake and toxicity of doxorubicin were investigated. An electrical field with a field strength of 500 $Vm^{-1}$ applied for a duration of 90 s resulted in neither reversible nor irreversible membrane breakdown as revealed by fluid phase uptake studies of the membrane impermeant tracer Lucifer yellow. However, treated spheroids showed an increased uptake of doxorubicin and, consequently, an increased toxicity following electrical field exposure. The electrical field raised intracellular reactive oxygen species (ROS) as revealed using 2′,7′-dichlorofluorescein diacetate ($H_2$DCFDA) as an indicator. ROS induced membrane lipid peroxidation since the lipid peroxidation end products malondialdehyde (MDA) and 4-hydroxy-2-(E)-nonenal (4-HNE) were detected after electrical field treatment. Moreover, lipid peroxidation decreased the lipid diffusion coefficient D from $4.2 \times 10^{-10}$ $cm^2$ $s^{-1}$ to $2.7 \times 10^{-10}$ $cm^2$ $s^{-1}$ in the control and treated sample, respectively, as revealed by fluorescence recovery after photobleaching (FRAP) experiments. The field effects could be mimicked by incubating spheroids with 100 nM hydrogen peroxide and were inhibited by the radical scavengers dehydroascorbate (DHA) and α-tocopherol (vitamin E), indicating that the increased uptake of doxorubicin after electrical field treatment is owing to lipid peroxidation and decreased membrane lipid mobility. Treatment of tumours with low intensity electrical fields may be useful to improve the cytotoxic capacity of anthracyclines.

**Keywords:** electrochemotherapy; electrical field; multicellular tumour spheroid; doxorubicin; lipid peroxidation; lipid diffusion

Electrochemotherapy (ECT) is a promising new approach to fighting cancer and has successfully been applied to regressing tumours in several animal models and clinical trials with squamous cell carcinomas, basal cell carcinomas, metastatic melanomas and liver tumours (Sersa et al, 1995; Glass et al, 1996; Heller et al, 1996; Mir et al, 1997; Ramirez et al, 1998, Belehradek et al, 1993). During ECT treatment, antineoplastic drugs with low cell membrane permeability, e.g. bleomycin (Poddevin et al, 1991), are delivered into cells by short and intense electric field pulses. These field pulses with a field strength of approximately 130 000 $Vm^{-1}$ are applied for microseconds and have been discussed to transiently increase the permeability of cell membranes by electroporation (Glass et al, 1996; Nishi et al, 1997). Owing to the high field strength of the applied electrical fields, pulses have to be administered locally at the solid tumour site of avoid serious side-effects of the electrical field on the patient. This implicates that the enhanced delivery of the anti-cancer agent is restricted to the area that has been electrically treated. Unlocalized tumours, however, remain untreated, making desirable an ECT approach that uses fairly lower field strengths which can be more generally applied to tissues or whole organs.

In the present study, we used multicellular prostate tumour spheroids of the DU-145 cell line to investigate whether treatment with low electrical field strengths increases the uptake and toxicity of the cell membrane-permeant anthracycline doxorubicin. Tumour spheroids were treated for 90 s with a single electrical field pulse of a field strength that has previously been shown to stimulate tumour cell proliferation (Sauer et al, 1997; Wartenberg et al, 1997). An experimental approach for ECT based on stimulation of cell proliferation should be promising since chemotherapeutics are known to be far more effective in rapid cycling as compared to slow-cycling tumour cells.

*Received 11 August 1998*
*Revised 20 November 1998*
*Accepted 27 November 1998*

*Correspondence to:* M Wartenberg

### MATERIALS AND METHODS

### Culture technique of multicellular tumour spheroids

The human prostate cancer cell line DU-145 was kindly provided by Dr J Carlsson, Uppsala, Sweden. Cells were cultured in Ham's F10 medium (Gibco, Live Technologies, Helgerman Court, MD, USA) as described previously (Sauer et al, 1997) Spheroids were grown from single cells (passages 2–15) seeded in 250 ml siliconated spinner flasks (Tecnomara, Fernwald, Germany) at $1 \times 10^5$ cells $ml^{-1}$. The spinner flask medium (175 ml) was stirred (20 rpm) using a stirrer system (Tecnomara) and partly changed every day. For the experiments, tumour spheroids of a size class of 150 ± 30 μm were used.

### Enzymatic dissociation of multicellular tumour spheroids

Dissociation of multicellular spheroids was performed as described previously (Wartenberg et al, 1998). In brief, spheroids were washed in phosphate-buffered saline (PBS) and placed in a

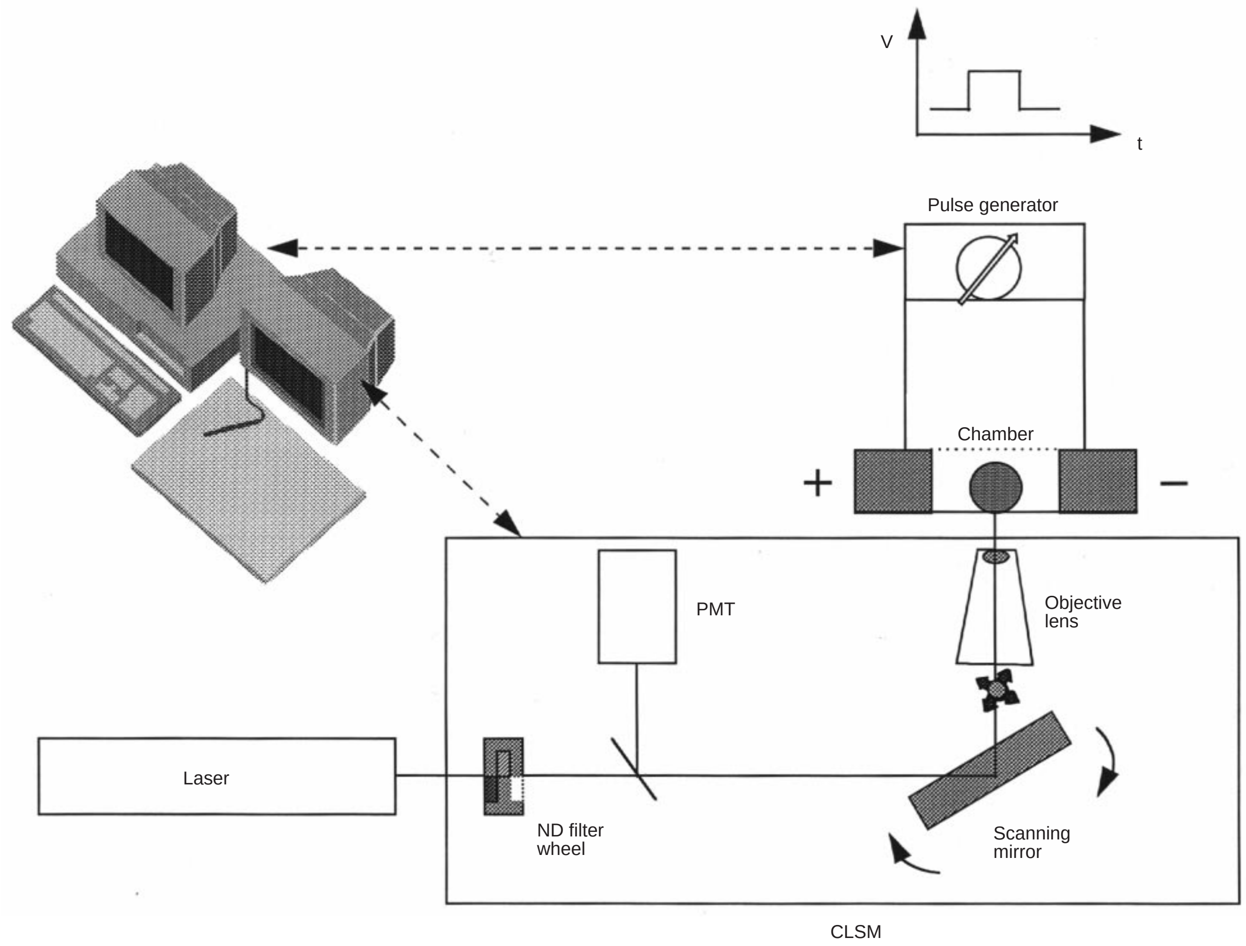

**Figure 1** Setup for electrical field treatment of multicellular tumour spheroids. Spheroids were placed into an incubation chamber filled with 'pulsing buffer'. The lateral walls of the chamber were formed by stainless (V4A) steel electrodes with an electrode distance of 2 mm and an electrode area of 0.4 $cm^2$. A computer-controlled pulse generator generated square wave pulses. During electrical field treatment the spheroids were examined by confocal laser scanning microscopy (CLSM). Fluorescence was recorded by photomultiplier tubes (PMT) and digital images were reconstructed by the CLSM image analysis software. By means of a filter changer (ND filter wheel) the laser beam scanning the tumour spheroids could be attenuated by a factor of approximately 1000. Flashes for photobleaching experiments could be applied within milliseconds

Petri dish containing 1 ml 0.2% Trypsin and 0.05% ethylenediaminetetraacetic acid (EDTA) in PBS. The spheroids were incubated for 5 min at 37°C and agitated every 30 s. Thereafter they were triturated and the enzymatic reaction was stopped by addition of 4 ml cell culture medium. After centrifugation at 80 ***g*** for 5 min the cells were resuspended in cell culture medium.

### Electrical field treatment

A schematic scheme of the experimental setup is depicted in Figure 1. For electropulse experiments, multicellular tumour spheroids were suspended in low ionic content, *N*-2-hydroxyethylpiperazine-N′-2-ethane-sulphonic acid (HEPES) (5 mM, pH 7.2) buffered 'pulsing medium' (Rols and Teissié, 1990) containing 255 mM sucrose, 1 mM calcium chloride and 1 mM magnesium chloride. The pulsing buffer had a conductivity of 500 μS $cm^{-1}$. Spheroids were placed into an incubation chamber between stainless (V4A) steel electrodes, with an electrode distance of 2 mm and an electrode area of 0.4 $cm^2$. The electrodes were connected to a custom-made voltage generator that generated square-wave voltage pulses. One single electric field pulse with a field strength of 500 $Vm^{-1}$ and a duration of 90 s was applied to the spheroids. The total current in the chamber amounted to 1 mA. The magnetic flux density in the proximity of the multicellular tumour spheroids was calculated to 0.079 μT, which is below the average laboratory noise level for low frequency electromagnetic fields (Cameron et al, 1993). After electric field treatment tumour spheroids were resuspended in F10 medium and incubated at 37°C for 90 min. In control experiments, we ensured that no water electrolysis, pH or temperature shifts occurred during the electropulse experiments.

### Doxorubicin uptake experiments and confocal laser scanning microscopy

For doxorubicin uptake experiments multicellular tumour spheroids were suspended in pulsing buffer supplemented with 4 μM doxorubicin (Sigma, Deisenhofen, Germany). Electropulsing in the presence of doxorubicin was performed under the optical control of an inverted confocal laser scanning microscope (CLSM 410, Carl Zeiss, Jena, Germany) equipped with an immersion corrected, 25 ×, numerical aperture 0.83, objective (Plan Neofluar, Carl Zeiss). Images of 512 × 512 pixels, 8 bits, were recorded

*© 1999 Cancer Research Campaign* 

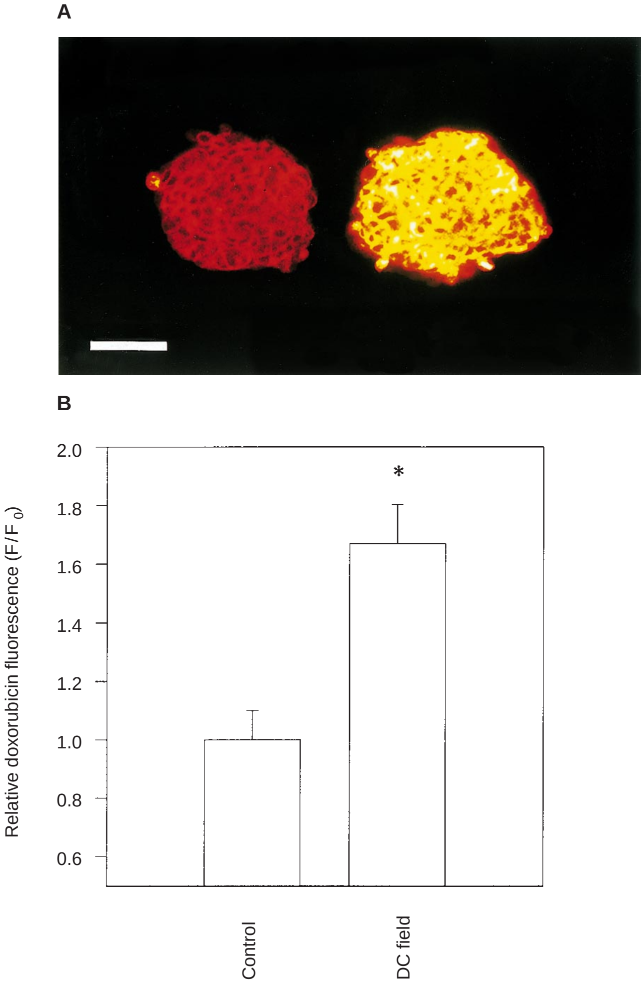

**Figure 2** Increased doxorubicin uptake in multicellular tumour spheroids treated with a single electrical field pulse (500 $Vm^{-1}$, 90 s) in the presence of 4 µM doxorubicin. (**A**) Doxorubicin distribution in representative spheroids; left side: untreated control spheroid; right side: electrical field treated spheroid. The images were recorded 90 min after electrical field application. The bar represents 100 µm. (**B**) Relative doxorubicin fluorescence in control and electrical field treated whole-mount multicellular spheroids 90 min after field application. The doxorubicin fluorescence in the control sample was set to 100%. About 30 spheroids in four independent experiments were used for the determination of each data point. *$P < 0.05$, significantly different from control every 60 s and were stored automatically to the host memory of the computer unit of the confocal setup. Image analysis was performed as described previously (Wartenberg et al, 1997). For the determination of doxorubicin retention, spheroids treated with electrical fields in the presence of 4 µM doxorubicin were transferred to F10 medium supplemented with 4 mM doxorubicin. Doxorubicin fluorescence was evaluated 90 min after addition of doxorubicin. Excitation of doxorubicin was performed using the 543-nm line of a helium-neon laser of the confocal setup. Emission was recorded using a longpass LP 570-nm filter set. In the experiments with free radical scavengers, tumour spheroids were pre-incubated for 2 h with either 2.5 mM dehydroascorbate (DHA), dissolved in pulsing buffer or for 24 h with 30 µM α-tocopherol (vitamin E), dissolved in dimethyl sulphoxide (DMSO). Final DMSO concentrations were < 0.1%.

### Determination of spheroid growth

Multicellular tumour spheroids were treated with a single electrical field pulse (500 $Vm^{-1}$, 90 s) either in the presence or absence of 4 µM doxorubicin; a third sample was treated with doxorubicin alone; and a negative control remained untreated. After 3 h, the spheroids were washed three times in F10 medium and further incubated for 3 days. Spheroid sizes were determined after washout of doxorubicin prior to the post-incubation period and subsequently after 24 and 72 h. Spheroid volumes were calculated according to $4/3 \times \pi \times r_s^2 \times r_l$, where $r_s$ represents the small radius of the respective spheroids and $r_l$ and large radius.

### Determination of fluid phase uptake and lethal staining of cells

For fluid phase uptake studies the hydrophilic, membrane-impermeant tracer Lucifer yellow CH (Sigma, Deisenhofen, Germany) was used. Either multicellular spheroids or single cells enzymatically dissociated from spheroids were suspended in pulsing buffer containing Lucifer yellow in a final concentration of 20 µM, and transferred to the pulsing chamber mounted to the stage of the confocal setup. Fluid uptake was continuously monitored before, during and after electrical field treatment with field strengths ranging from 500 $Vm^{-1}$ to 3000 $Vm^{-1}$, applied for 90 s. Reversible and irreversible membrane breakdown was evaluated as intracellular increase of Lucifer yellow fluorescence. Excitation was performed using the 488-nm line of the argon-ion laser of the confocal setup. Emission was recorded using a longpass LP515-nm filter set.

Lethal staining was performed using the lethal cell marker Ethidium homodimer-1 (Ethd-1) (Molecular Probes, Eugene, OR, USA). Spheroids were treated with a field strength of 3000 $Vm^{-1}$ for 90 s and were incubated 15 min subsequent to electrical field treatment for 30 min at room temperature with Ethd-1 (final concentration 12 µM). Excitation of Ethd-1 was performed using the 488-nm line of the argon-ion laser. Emission was recorded using a longpass LP590-nm filter set.

### Determination of intracellular redox state

Intracellular redox state levels were measured using the fluorescent dye 2′,7′-dichlorofluorescein diacetate ($H_2$DCFDA) (Molecular Probes), which is a non-polar compound that is converted into a non-fluorescent polar derivative by cellular esterases after incorporation into cells. $H_2$DCF is membrane impermeable and is rapidly oxidized to the highly fluorescent 2′,7′-dichlorofluorescein (DCF) in the presence of intracellular ROS (Frenkel and Gleichauf, 1991). For the experiments, multicellular tumour spheroids were incubated in F10 medium containing 20 µM $H_2$DCFDA for 30 min at room temperature. Spheroids were then washed twice in F10 medium, once in pulsing buffer and transferred to the experimental chamber. For fluorescence excitation, the 488-nm band of the argon ion laser of the confocal setup was used. Emission was recorded using a longpass LP515-nm filter set.

### Determination of lipid peroxidation

Lipid peroxidation of membrane preparations was measured by use of a colorimetrically based kit (Calbiochem-Novabiochem

 © 1999 Cancer Research Campaign

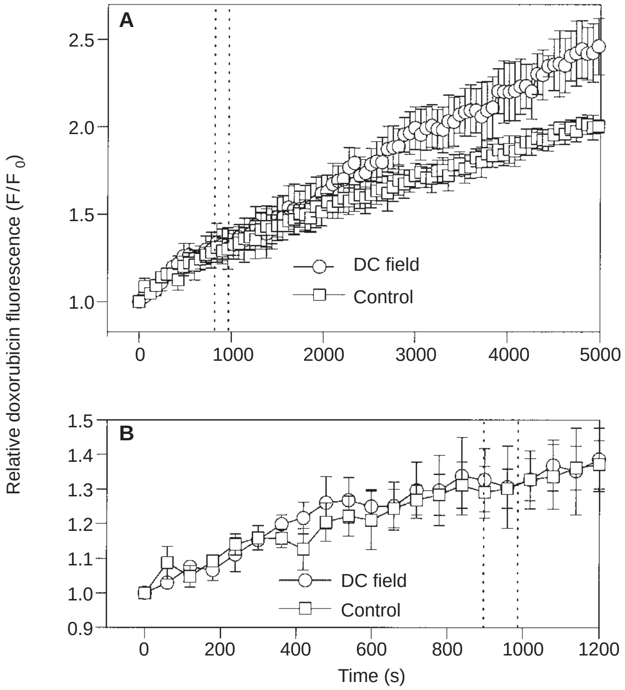

**Figure 3** Time course of doxorubicin uptake in multicellular spheroids. (**A**) Doxorubicin fluorescence increase prior, during and following electrical field (500 Vm$^{-1}$, 90 s) treatment. (**B**) Presentation of doxorubicin fluorescence increase during electrical field treatment. The electrical field was applied during the time indicated by the vertical dashed lines. Note that doxorubicin uptake is not enhanced during electrical field application, indicating that the used field strength did not induce membrane breakdown processes.

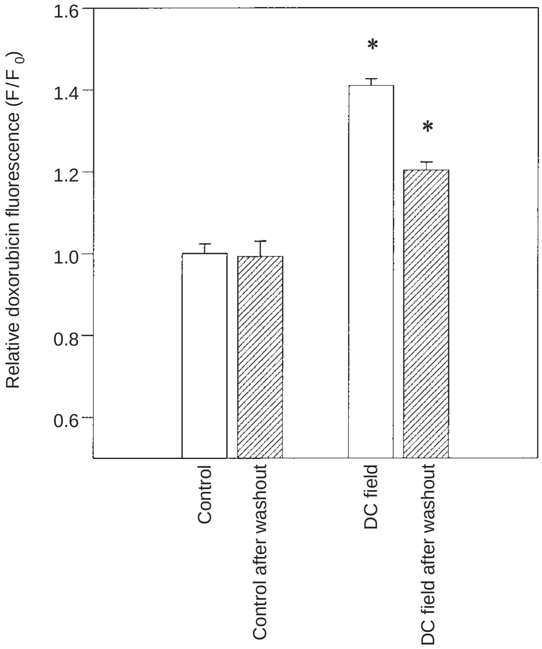

**Figure 4** Doxorubicin retention in electrical field treated (500 Vm$^{-1}$, 90 s) multicellular spheroids. Electrical field treated spheroids (hatched bars) and an untreated control (open bars) were incubated with 4 μM doxorubicin during electrical field application and post-incubated in the presence of doxorubicin for 90 min. Thereafter, external doxorubicin was washed out and doxorubicin fluorescence was evaluated following an incubation period of 90 min in doxorubicin-free F10 medium. About 30 spheroids in three independent experiments were used for the determination of each data point. *$P < 0.05$, significantly different from control

International, Inc. San Diego, CA, USA). In brief, membrane preparations of $10^5$ cells containing end products of lipid peroxidation, e.g. malonaldehyde and 4-hydroxy-2(E)-nonenal, were reacted with the chromophore *N*-methyl-2-phenylindole and with methanesulphonic acid (40 min at 45°C). The samples were cooled on ice and centrifuged at 10 000 ***g*** for 5 min at room temperature. The absorbance of the supernatant at 590-nm and 25°C was read on a spectrofluorometer. The combined concentration of malondialdehyde and 4-hydroxy-2(E)-nonenal was determined from a standard curve.

### Fluorescence after photobleaching (FRAP) experiments

FRAP experiments were performed using the line-scanning microphotolysis technique recently developed by Wedekind et al (1996). In brief, electrical field (500 Vm$^{-1}$, 90 s)-treated and untreated control multicellular tumour spheroids were labelled 2 h after field exposure for 15 min with the fluorescent lipid analogue 2-(6-(7-nitrobenz-2-oxa-1,3-diazol-4-yl)amino)hexanoyl-1-hexadecanoyl-*sn*-glycero-3-phosphocholine (NBD-$C_6$-HPC) (Molecular Probes) dissolved in a final concentration of 10 μg ml$^{-1}$ in E1 buffer containing 135 mM sodium chloride, 5.4 mM potassium chloride, 1.8 mM calcium chloride, 1 mM magnesium chloride, 10 mM glucose, 10 mM HEPES (pH 7.4 at 23°C). They were subsequently washed three times in E1 buffer and transferred to an incubation chamber mounted on the stage of the confocal setup. The initial fluorescence before photobleaching was evaluated by recording a 512 × 512 pixel *x,y* scan image. From this prebleach image the mean fluorescence was determined in the region of interest predisposed for photobleaching. The confocal microscope was subsequently switched to the line scan mode and the laser power was increased from 2.5 mW to 25 mW. During line scanning, which was performed for 3 s, the laser beam was repetitively scanned at high speed (3 ms per line scan) along a selected line. After photolysis along the selected line, full frame images (512 × 512 pixel) were recorded in intervals of 15 s, and the fluorescence recovery in the centre of the line was determined using an overlay mask. The data were plotted for display by non-linear least-squares fit of the data and the lipid lateral diffusion coefficient was calculated from the recovery curves according to Koppel et al (1979) who described the relationship between fluorescence F at the scanning point Δx from the centre of the bleached area at time *t* by the following equations

$$F(\Delta x,t) = F_0\{[1-\beta\alpha_2(t)]\exp[-(\Delta x)^2/\omega^2(t)]\} - \{(1-\beta)\alpha_0\exp[-(\Delta x)^2/\omega_0^2]\} \quad (1)$$

$$\alpha_2 = \alpha_0/(1 + t/\tau_D) \quad (2)$$

$$\tau_D = \omega_0^2/4D \quad (3)$$

where β represents the size of the mobile fraction, $\alpha_0$ is a constant that characterizes the extent of bleaching, $\omega_0$ is the $1/e$ radius of the laser beam size, $\tau_D$ is the diffusion time, and $D$ is the diffusion coefficient.

### Statistical analysis

Data are given as mean values ± s.e.m. with *n* denoting the number of experiments. Student's *t*-test for unpaired data was applied as appropriate. A value of $P < 0.05$ was considered significant.

© 1999 Cancer Research Campaign 

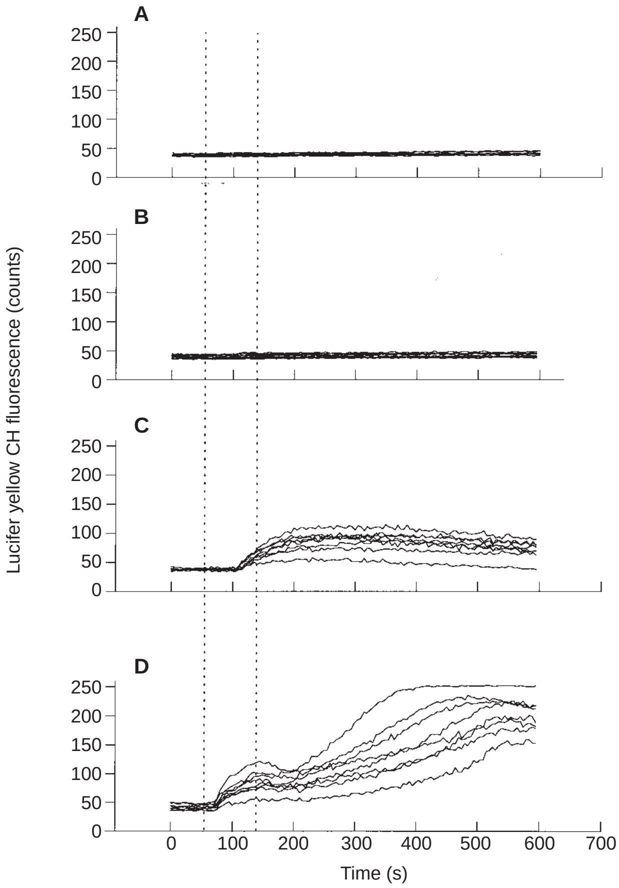

**Figure 5** Time course of fluid phase uptake of Lucifer yellow CH in single cells treated with different field strengths. Single cells were enzymatically dissociated from multicellular tumour spheroids. (**A**) Control; (**B**) 500 $Vm^{-1}$, 90 s; (**C**) 1500 $Vm^{-1}$, 90 s; (**D**) 2000 $Vm^{-1}$, 90 s. The electrical field was applied during the time indicated by the vertical dashed lines

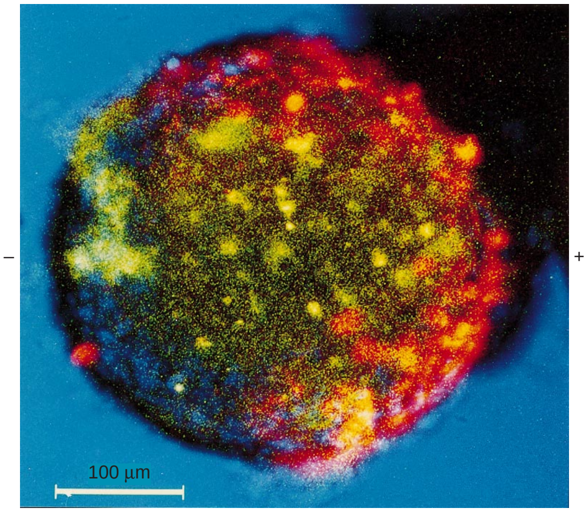

**Figure 6** Lucifer yellow CH fluid phase uptake and Ethd-1 lethal staining in a representative multicellular tumour spheroid treated with a 3000 $Vm^{-1}$, 90 s electrical field. Shown is a false colour overlay of a transmission image (blue), a Lucifer yellow fluorescence image (green), and a Ethd-1 fluorescence image (red). Note that cell lethality is increased at the anode-facing side of the spheroid. The bar represents 100 µm

### RESULTS

### Enhanced doxorubicin uptake and retention in multicellular tumour spheroids treated with electrical fields

When multicellular tumour spheroids were treated with a single electrical field pulse with a field strength of 500 $Vm^{-1}$ and a duration of 90 s, which has previously been shown to stimulate tumour growth (Sauer et al, 1997; Wartenberg et al, 1997), a significant increase by 67 ± 3% of doxorubicin uptake (Figure 2 A,B) was observed after 90 min ($n$ = 4). Doxorubicin fluorescence was homogenously distributed within the tissue, i.e. no preferential accumulation at the cathode- or anode-facing was observed. The time course of doxorubicin uptake was monitored by CLSM during electrical field treatment. Figure 3 A,B shows that doxorubicin uptake is not enhanced during, but following, the electrical field pulse, indicating that the observed effect is not owing to either reversible or irreversible membrane breakdown ($n$ = 3). The observed increase in doxorubicin uptake after electrical field treatment may be at least partially owing to reduced doxorubicin efflux. Therefore, doxorubicin retention was assessed in electrical field-treated tumour spheroids 90 min after doxorubicin removal from the external medium. Figure 4 shows that in the electrical field-treated sample a significant efflux of doxorubicin after washout of external doxorubicin occurred. This indicates that the observed enhancement of doxorubicin retention following electrical field treatment may be owing to physical changes in membrane permeability which affect both efflux and influx kinetics of doxorubicin. However, doxorubicin retention after washout of external doxorubicin was still about 20% enlarged in the electrical field treated sample as compared to control ($n$ = 3) which presumably results from intracellular binding of the compound.

To exclude that either reversible or irreversible membrane breakdown occurred during and after electrical field treatment, spheroids and single cells enzymatically dissociated from spheroids were treated with different field strengths in the presence of the polar fluorescence dye Lucifer yellow CH. Membrane breakdown processes should result in an increased uptake of this fluid phase marker. Figure 5 demonstrates that no fluid phase uptake of Lucifer yellow CH occurred when field strengths of 500 $Vm^{-1}$ and 1000 $Vm^{-1}$ (not shown) were applied for 90 s. A marked fluid phase uptake owing to electrical field treatment was observed with a field strength of 1500 $Vm^{-1}$. The Lucifer yellow uptake occurred during electrical field application and remained on a sustained plateau, indicating reversible membrane breakdown ($n$ = 3). Treatment with a field pulse of 2000 $Vm^{-1}$ resulted in fluid phase uptake during field application, followed by a further rise of Lucifer yellow fluorescence shortly after treatment, which is indicative for irreversible membrane breakdown ($n$ = 3). When multicellular tumour spheroids were treated with an electrical field pulse of 3000 $Vm^{-1}$ and Lucifer yellow staining was co-localized with Ethd-1 staining of lethal cells, an increased cell lethality was observed at the anode-facing pole of the spheroid, indicating more pronounced cell membrane potential changes resulting in irreversible membrane breakdown processes (Figure 6) ($n$ = 3).

### Doxorubicin toxicity is increased after DC electrical field treatment of multicellular tumour spheroids

To test whether adjuvant DC electrical field treatment (500 $Vm^{-1}$, 90 s) augmented doxorubicin toxicity, multicellular tumour spheroids were electrical field-treated in the presence of doxorubicin, and

 © 1999 Cancer Research Campaign

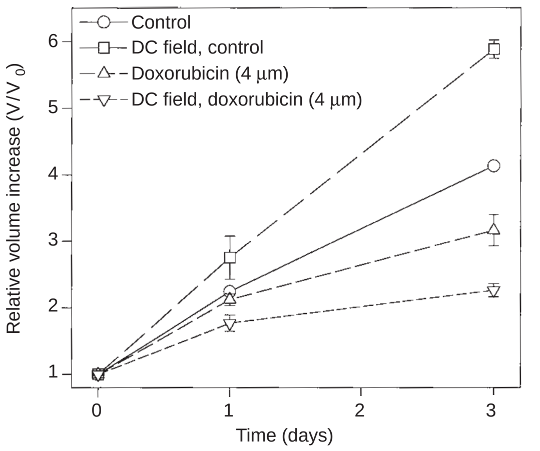

**Figure 7** Growth kinetics of multicellular tumour spheroids after electrical field (500 $Vm^{-1}$, 90 s) treatment. One sample of spheroids was treated with a single electrical field pulse (500 $Vm^{-1}$, 90 s) in the presence of doxorubicin (4 µM) (▽). A second sample was electrical field treated in the absence of doxorubicin (□). A third sample was treated with doxorubicin (4 µM) alone (△). A control sample remained untreated (○). Note that electrical field treatment in the absence of doxorubicin enhanced the growth kinetics of tumour spheroids as previously reported (Sauer et al, 1997)

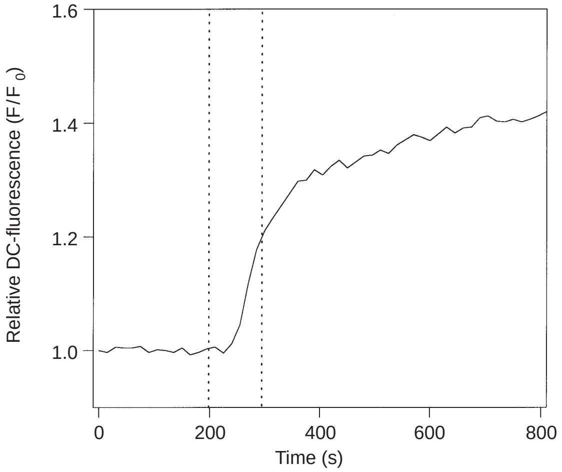

**Figure 8** ROS generation during and following treatment of multicellular tumour spheroids with a single electrical field pulse (500 $Vm^{-1}$, 90 s). Representative trace of normalized dichlorofluorescein (DCF) fluorescence. The electrical field was applied during the time indicated by the dashed lines spheroid growth was monitored. Figure 7 shows the growth kinetics of control spheroids, spheroids treated with doxorubicin alone and spheroids treated with a single electrical field pulse (500 $Vm^{-1}$, 90 s) in the presence or absence of doxorubicin. As reported previously (Sauer et al, 1997; Wartenberg et al, 1997), the electrical field pulse led to an enhancement of tumour growth. However, electrical field treatment in the presence of doxorubicin resulted in a significantly decreased growth kinetics as compared to spheroids treated with doxorubicin alone ($n = 3$).

### The increased doxorubicin uptake is owing to the generation of intracellular reactive oxygen species by electrical field treatment

To investigate underlying mechanisms for the observed increase of doxorubicin uptake following electrical field treatment, intracellular

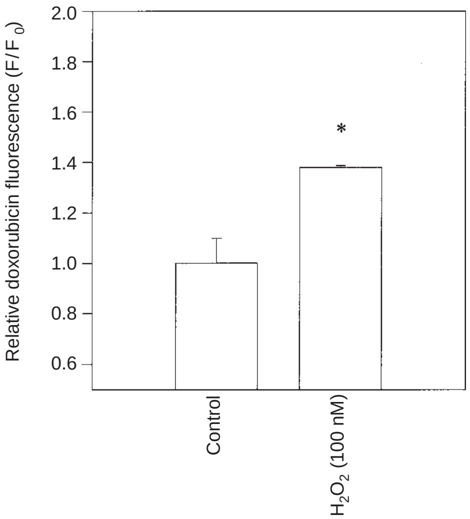

**Figure 9** Effect of 100 nM hydrogen peroxide on the doxorubicin uptake in multicellular spheroids. Spheroids were treated for 90 min with $H_2O_2$ in the presence of doxorubicin and doxorubicin fluorescence was evaluated in whole-mount spheroids. About 30 spheroids in four independent experiments were used for the determination of each data point. *$P < 0.05$, significantly different from control

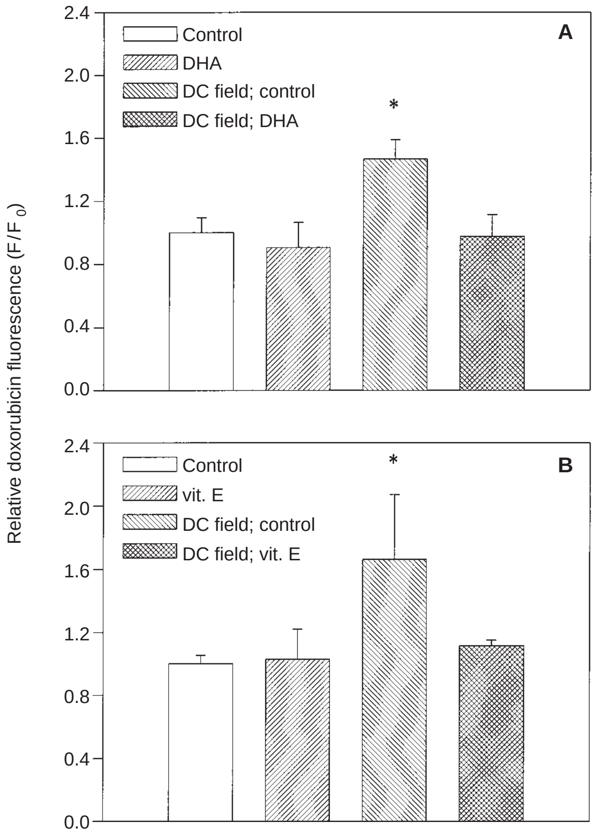

**Figure 10** Effects of the radical scavengers DHA (2.5 mM) (**A**) and α-tocopherol (vitamin E) (30 µM) (**B**) on doxorubicin uptake following electrical field (500 $Vm^{-1}$, 90 s) treatment. Doxorubicin fluorescence was determined after an incubation period of 90 min in: (1) an untreated control; (2) an untreated control preincubated for 2 h and 24 h with DHA and α-tocopherol respectively; (3) an electrical field treated sample; (4) in an electrical field treated sample after preincubation with radical scavengers. About 30 spheroids in three independent experiments were used for the determination of each data point. *$P < 0.05$, significantly different from control

*© 1999 Cancer Research Campaign*

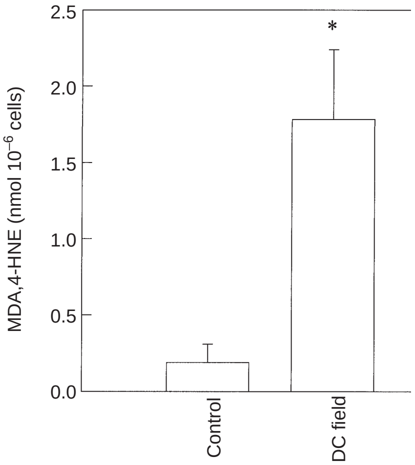

**Figure 11** Determination of the lipid peroxidation end-products 4-HNE and MDA in cell extracts from multicellular tumour spheroids treated with a single electrical field pulse (500 $Vm^{-1}$, 90 s). Evaluation of lipid peroxidation was performed 2 h after electrical field treatment. *$P < 0.05$, significantly different from control reactive oxygen species (ROS) were determined using DCF as an indicator. Figure 8 shows that during electrical field treatment ROS were generated ($n$ = 5). The amount of ROS generated was equivalent to the DCF signal elicited with 50–100 nM hydrogen peroxide (Wartenberg et al, 1997). Consequently, nanomolar concentrations (100 nM) of externally added hydrogen peroxide mimicked the effect of the electrical field on doxorubicin uptake (see Figure 9), indicating that ROS generation subsequent to electrical field treatment is sufficient to enhance doxorubicin uptake ($n$ = 4). This was further corroborated by experiments where multicellular tumour spheroids were preincubated with the radical scavengers dehydroascorbate (DHA) (2.5 mM) and α-tocopherol (vitamin E) (30 μM). Figure 10 A,B clearly demonstrates that both DHA and vitamin E (dissolved in DMSO) present during electrical field treatment reduced doxorubicin uptake to the control level of the untreated sample ($n$ = 5), indicating that ROS generation following electrical field treatment of multicellular tumour spheroids is mediating the enhancement of doxorubicin uptake. DMSO in the absence of α-tocopherol did not exert any effect on the electrical field-induced doxorubicin accumulation (data not shown).

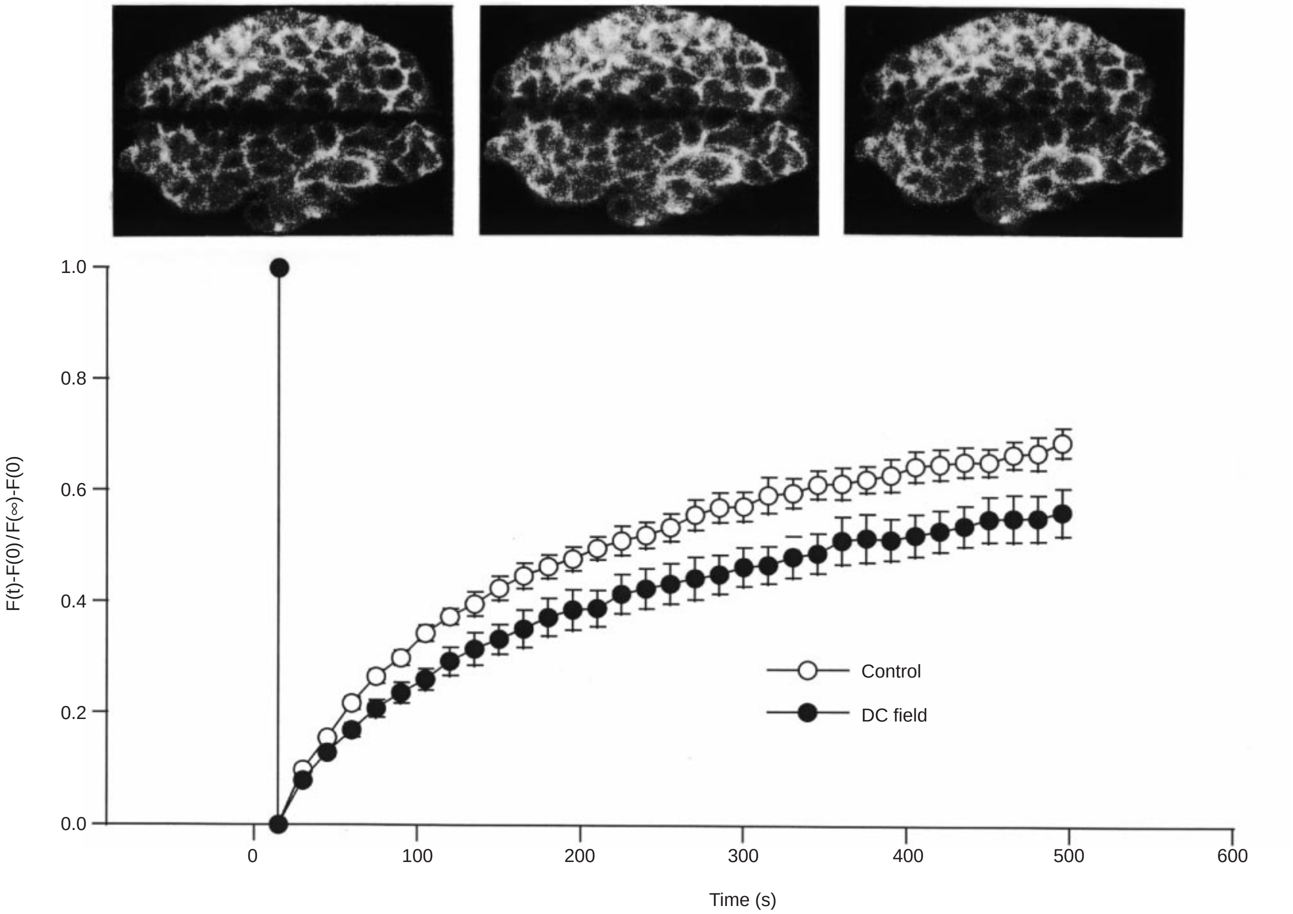

**Figure 12** Lipid lateral diffusion is decreased in multicellular tumour spheroids treated with a single electrical field pulse (500 $Vm^{-1}$, 90 s). FRAP experiments were performed with spheroids stained with the fluorescent lipid analogue NBD-$C_6$-HPC. Photobleaching along a preselected line was achieved by using the line-scanning mode of the confocal microscope. The fluorescence images show (from the left to the right) the fluorescence recovery along the photobleached line in a representative tumour spheroid 0 s, 150 s and 300 s after photobleaching. The traces show one of four experiments with comparable results. The fluorescence recovery of at least three spheroids was determined to give one data point

 *© 1999 Cancer Research Campaign*

### ROS generated during electrical field treatment of multicellular tumour spheroids induce membrane lipid peroxidation

ROS generated during electrical field treatment of multicellular tumour spheroids may induce membrane lipid peroxidation, resulting in an alteration of the permeability properties of the cell membrane for doxorubicin. To assess lipid peroxidation malondialdehyde (MDA) and 4-hydroxy-2(E)-nonenal (4-HNE), which are end products of lipid peroxidation, were evaluated in cell extracts of electrical field treated multicellular spheroids 2 h after electrical field treatment ($n = 3$). Figure 11 shows that MDA and 4-HNE in a concentration of $1.78 \pm 0.26$ nmol $10^{-6}$ cells were generated following electrical field treatment, indicating membrane lipid peroxidation, which presumably resulted in changes of lipid bilayer structure mediating increased doxorubicin uptake.

### Electrical field-induced membrane rigidization

Lipid peroxidation following electrical field treatment of multicellular tumour spheroids may alter the fluidity of cell membranes, which may have effects on doxorubicin uptake and efflux kinetics. To evaluate electrical field-induced changes in lipid mobility FRAP experiments were performed using the fluorescent lipid analogue NBD-$C_6$-HPC and the line-scanning mode of the CLSM. Our data show that fluorescence recovery after photobleaching was reduced (Figure 12) when multicellular tumour spheroids had been treated with an electrical field pulse 2 h prior to the FRAP experiments, which resulted in a significantly decreased diffusion coefficient D with $4.2 \times 10^{-10} \pm 1 \times 10^{-10}$ cm$^2$ s$^{-1}$ as compared to $2.7 \times 10^{-10} \pm 0.6 \times 10^{-10}$ cm$^2$ s$^{-1}$ in the untreated control group ($n = 13$ spheroids from four different spheroid cultures).

### DISCUSSION

The electrical field strengths used in current approaches of ECT are markedly above the critical field strengths for reversible or irreversible membrane breakdown (Domenge et al, 1996; Heller et al, 1997). Therefore, the enhancement of drug delivery following electrical field treatment is thought to be mediated by electroporation processes (Belehradek et al, 1994). Owing to severe side-effects of the electrical fields on non-cancerous cells, the applicability of ECT is reduced to the close vicinity of tumours.

The non-lethal low field strength (500 Vm$^{-1}$, 90 s) applied in the present study has previously been used by us to enhance the growth kinetics of multicellular tumour spheroids and to recruit quiescent cells in the depth of the tumour tissue for the cell cycle (Sauer et al, 1997; Wartenberg et al, 1997). We have chosen this proliferation-inducing electrical field strength since proliferation-active cells have been demonstrated to be more susceptible towards treatment with anticancer agents than dormant cells, which have been reported to display features of resistance both towards radiation and chemotherapy (Remvikos et al, 1993; Olive and Durand, 1994; Hietanen et al, 1995; Wartenberg et al, 1998). An approach using low field-strength non-toxic electrical fields for ECT should be promising and may result in an increased efficacy of chemotherapy. However, chemosensitization by stimulation of tumour cell proliferation should assure the best possible access of administered cytostatics to the tumours in order to avoid a fatal ECT-induced enhancement in tumour growth.

The data of the present study demonstrate that treatment of multicellular tumour spheroids with a single electrical field pulse of low field-strength (500 Vm$^{-1}$, 90 s) significantly increased doxorubicin uptake and, consequently, doxorubicin toxicity. We could show that the applied electrical field strength did not lead to either reversible or irreversible membrane breakdown processes since – as described previously (Sauer et al, 1997) – a field strength of 500 Vm$^{-1}$ applied to a spherical object of approximately 150 μm in diameter resulted in a membrane potential change of 75 mV, which is far below the critical membrane potential change of 0.5–1.5 V that has been reported to be essential to induce membrane breakdown processes (Weaver, 1993). Moreover, fluid phase uptake studies with the hydrophilic fluorescence probe Lucifer yellow CH showed that fluid phase uptake was not observed in single cells when field strengths of 500 Vm$^{-1}$ (90 s) were applied. Experiments with different field strengths revealed that reversible membrane breakdown occurred with a critical electrical field strength of 1500 Vm$^{-1}$ (90 s), which resulted in a sustained rise in Lucifer yellow uptake during and shortly following electrical field. Irreversible membrane breakdown was observed at a field strength of 2000 Vm$^{-1}$ (90 s) and was characterized by an initial rise of Lucifer yellow fluorescence during electrical field treatment, which was followed by a longlasting fluid phase uptake. As previously described (Sauer et al, 1997), lethal effects of the electrical field were found with a critical field strength of 3000 Vm$^{-1}$. Interestingly, cell lethality as indicated by Ethd-1 lethal cell staining was mainly observed at the anode-facing pole of the spheroid. This effect can be explained by a superimposition of the electrical field vector of the cell membrane potential and the electrical field vector of the external electrical field, which are acting in the same direction and lead to enlarged membrane potential changes at the anode-facing as compared to the cathode-facing side of the spheroid (Sauer et al, 1997).

Electrical fields have been shown to change the intracellular redox state (Wartenberg et al, 1997). An increase in intracellular ROS may lead to lipid peroxidation and to altered permeability properties of the cell membrane (Stanimirovic et al, 1995). Changes in the intracellular redox state occurred during electrical field treatment with a 500 Vm$^{-1}$ (90 s) electrical field using the fluorescence indicator DCF. Furthermore, the lipid peroxidation products MDA and 4-HNE were detected in the cell homogenates of tumour spheroids that had been treated with 500 Vm$^{-1}$ (90 s) electrical fields. To investigate whether electrical field generated ROS were a prerequisite for the enhancement of doxorubicin uptake, spheroids were incubated in the absence of an external electrical field with 100 nM hydrogen peroxide, which resulted in a doxorubicin uptake not significantly different from the uptake observed after electrical field treatment, indicating that the electrical field-generated ROS were equivalent to 100 nM externally added hydrogen peroxide, as previously reported (Wartenberg et al, 1997). Moreover, incubation of multicellular tumour spheroids with DHA, a radical scavenger of cytoplasmic ROS, and α-tocopherol, which inhibits lipid peroxidation by integration into the plasma membrane (Susa et al, 1996), abolished the electrical field-induced enhancement of doxorubicin uptake, indicating that the observed effect was owing to changes in the cellular oxidized state.

The data of the present study suggest that lipid peroxidation following electrical field treatment results in an increased membrane permeability for doxorubicin, which affects both the uptake and the efflux of the drug, but as a net effect results in increased doxorubicin retention, presumably owing to intracellular binding. It has been previously shown that lipid peroxidation results in membrane rigidization with the consequence of altered permeation properties of the cell membrane (Deliconstantinos et

© 1999 Cancer Research Campaign 

al, 1996; Garcia et al, 1997; Verstraeten et al, 1997). These data were corroborated by the FRAP experiments of the present study, which show that electrical field treatment of tumour spheroids significantly decreased membrane fluidity, presumably owing to the ROS generated by electrical field treatment.

The parameters that govern doxorubicin uptake in cells are still a matter of debate. It has been shown that doxorubicin binds to anionic phospholipids in the membrane which involves electrostatic interaction as well as penetration of the electrostatically bound drug between the acyl chains (de Wolf et al, 1993; Speelmans et al, 1994). The uncharged form of the drug is transported via passive diffusion, of which the driving force is the intracellular acid pH and drug binding to intracellular targets (Harrigan et al, 1993). In the cytoplasmic compartment, doxorubicin has been demonstrated to generate ROS by doxorubicin quinone reduction and redox-cycling (Keizer et al, 1990).

Significant scientific efforts have been undertaken to investigate the cytotoxic effects of ROS generated by doxorubicin redox-cycling, which have been related to ROS-dependent DNA damage and lipid peroxidation with the consequence of impaired signal transduction pathways (Keizer et al, 1990; Cummings et al, 1992). However, the influence of the intracellular oxidized state and lipid peroxidation on doxorubicin uptake is not well-documented. In a recent study, doxorubicin toxicity was found to be enhanced after incubation of breast cancer cells with polyunsaturated fatty acids, which are known to induce lipid peroxidation. Incubation with radical scavengers caused cell viability to return to the baseline level of doxorubicin in the absence of polyunsaturated fatty acids (Germain et al, 1998). Moreover, in a doxorubicin-resistant mammary tumour cell line, lipid peroxidation levels were found to be lower than in the wild-type cell line (Benchekroun et al, 1993). These data point towards the notion that lipid peroxidation may be a general mechanism by which the permeability of tumour cell membranes may be increased for doxorubicin and its anthracycline derivatives.

The ECT approach elaborated in the present study for the in vitro model of multicellular tumour spheroids demonstrates that electrical field strengths far below the threshold of membrane breakdown processes effectively increase doxorubicin uptake in the tumour tissue. Changing the intracellular oxidized state of tumours, or tumour-bearing organs, by low field-strength electrical fields may be useful to chemosensitize tumours for chemotherapeutic treatment. Since electrical fields can be applied locally, an increased doxorubicin toxicity in the target area may be achieved with lower doses of orally or intravenously administered drug as are currently applied during conventional chemotherapy.

The in vitro approach of the present study may initiate in vivo investigations with the aim of selectively increasing anthracycline uptake in the diseased organ rather than the whole body, which – when lower doses of the cytostatic are applied – should result in less severe side-effects for the patient.

### ACKNOWLEDGEMENTS

This work is part of the MD thesis of Volker Pütz. We gratefully acknowledge support by the Köln Fortune Programm, University of Cologne, Germany.

### REFERENCES

Belehradek M, Domenge C, Luboinski B, Orowski S, Belehradek J Jr and Mir LM (1993) Electrochemotherapy, a new antitumor treatment. First clinical phase I–II trial. *Cancer* **72**: 3694–3700

Belehradek J Jr, Orlowski S, Ramirez LH, Pron G, Poddevin B and Mir LM (1994) Electropermeabilization of cells in tissues assessed by qualitative electroloading of bleomycin. *Biochim Biophys Acta* **1190**: 155–163

Benchekroun NM, Pourquier P, Schott B and Robert J (1993) Doxorubicin-induced lipid peroxidation and glutathione peroxidase activity in tumor cell lines selected for resistance to doxorubicin. *Eur J Biochem* **211**: 141–146

Cameron IL, Hardman WE, Winters WD, Zimmerman S and Zimmerman AM (1993) Environmental magnetic fields: influences on early embryogenesis. *J Cell Biochem* **51**: 417–425

Cummings J, Willmott N, Hoey BM, Marley ES and Smyth JF (1992) The consequences of doxorubicin quinone reduction in vivo in tumour tissue. *Biochem Pharmacol* **44**: 2165–2174

de Wolf FA, Staffhorst RW, Smits HP, Onwezen MF and Kruijff B (1993) Role of anionic phospholipids in the interaction of doxorubicin and plasma membrane vesicles: drug binding and structural consequences in bacterial systems. *Biochemistry* **32**: 6688–6695

Deliconstantinos G, Villiotou V and Stavrides JC (1996) Tumour promotor tert-butyl-hydroperoxide induces peroxynitrite formation in human erythrocytes. *Anticancer Res* **16**: 2969–2979

Domenge C, Orlowski S, Luboinski B, De Baere T, Schwaab G, Belehradek J Jr and Mir LM (1996) Antitumor electrochemotherapy: new advances in the clinical protocol. *Cancer* **77**: 956–963

Frenkel K and Gleichauf C (1991) Hydrogen peroxide formation by cells treated with a tumour promotor. *Free Rad Res Comms* **12–13**: 783–794

Garcia JJ, Reiter RJ, Guerrero JM, Escames G, Yu BP, Oh CS and Munoz-Hoyos A (1997) Melatonin prevents changes in microsomal membrane fluidity during induced lipid peroxidation. *FEBS Lett* **26**: 297–300

Germain E, Chajes V, Cognault S, Lhuillery C and Bougnoux P (1998) Enhancement of doxorubicin toxicity by polyunsaturated fatty acids in the human breast tumor cell line MDA-MB-231: relationship to lipid peroxidation. *Int J Cancer* **75**: 578–583

Glass LF, Pepine ML, Fenske NA, Jaroszeski M, Reintgen DS and Heller R (1996) Bleomycin-mediated electrochemotherapy of metastatic melanoma. *Arch Dermatol* **132**: 1353–1357

Harrigan PR, Wong KF, Redelmeier TE, Wheeler JJ and Cullis PR (1993) Accumulation of doxorubicin and other lipophilic amines into large unilamellar vesicles in response to transmembrane pH gradients. *Biochim Biophys Acta* **1149**: 329–338

Heller R, Jaroszeski MJ, Glass LF, Messina JL, Rapaport DP, DeConti RC, Fenske NA, Gilbert RA, Mir LM and Reintgen DS (1996) Phase I/II trial for the treatment of cutaneous and subcutaneous tumours using electrochemotherapy. *Cancer* **77**: 964–971

Heller R, Jaroszeski M, Perrot R, Messina J and Gilbert R (1997) Effective treatment of B16 melanoma by direct delivery of bleomycin using electrochemotherapy. *Melanoma Res* **7**: 10–18

Hietanen P, Blomqvist C, Wasenius VM, Niskanen E, Frarnssila K and Nordling S (1995) Do DNA ploidy and S-phase fraction in primary tumour predict the response to chemotherapy in metastatic breast cancer? *Br J Cancer* **71**: 1029–1032

Keizer HG, Pinedo HM, Schuurhuist GJ and Joenje H (1990) Doxorubicin (adriamycin): a critical review of free radical-dependent mechanisms of cytotoxicity. *Pharmac Ther* **47**: 219–231

Koppel DE (1979) Fluorescence redistribution after photobleaching. *Biophys J* **28**: 281–292

Mir LM, Devauchelle P, Quintin-Colonna F, Delisle F, Dolinger S, Fradelizi D, Belehradek J Jr and Orlowski S (1997) First clinical trial of cat soft-tissue sarcomas treatment by electrochemotherapy. *Br J Cancer* **76**: 1617–1622

Nishi T, Dev SB, Yoshizato K, Kuratsu J and Ushio Y (1997) Treatment of cancer using pulsed electric field in combination with chemotherapeutic agents or genes. *Human Cell* **10**: 81–86

Olive PL and Durand RE (1994) Drug and radiation resistance in spheroids: cell contact and kinetics. *Cancer Metastas Rev* **13**: 121–138

Poddevin B, Orlowski S, Belehradek J Jr and Mir LM (1991) Very high cytotoxicity of bleomycin introduced into the cytosol of cells in culture. *Biochem Pharmacol* **42**: S67–S75

Ramirez LH, Orlowski S, An D, Bindloula G, Dzodic R, Ardouin P, Bognel C, Belehradek J Jr, Munck J-N and Mir LM (1998) Electrochemotherapy on liver tumours in rabbits. *Br J Cancer* **77**: 2104–2111

Remvikos Y, Mosseri V, Zajdela A, Fourquet A, Durand JC, Pouillard P and Magdelenat H (1993) Prognostic value of the S-phase fraction of breast cancers treated by primary radiotherapy or neoadjuvant chemotherapy. *Ann NY Acad Sci* **698**: 193–203

Rols M-P and Teissié (1990) Electropermeabilization of mammalian cells. *Biophys J* **58**: 1089–1098

 © 1999 Cancer Research Campaign

Sauer H, Hescheler J, Reis D, Diedershagen H, Niedermeier W and Wartenberg M (1997) DC electrical field-induced c-*fos* expression and growth stimulation in multicellular prostate cancer spheroids. *Br J Cancer* **75**: 1481–1488

Sersa G, Cemazar M and Miklavcic D (1995) Antitumor effectiveness of electrochemotherapy with cis-diamminedichloroplatinum (II) in mice. *Cancer Res* **55**: 3450–3455

Speelmans G, Staffhorst RW, de Kruijff B and de Wolf FA (1994) Transport studies of doxorubicin in model membranes indicate a difference in passive diffusion across and binding at the outer and inner leaflets of the plasma membrane. *Biochemistry* **33**: 13761–13768

Stanimirovic DB, Wong J, Ball R and Durkin JP (1995) Free radical-induced endothelial membrane disfunction at the site of blood-brain barrier: relationship between lipid peroxidation, Na,K-ATPase activity, and $^{51}Cr$ release. *Neurochem Res* **20**: 1417–1427

Susa N, Ueno S, Furukawa Y and Sugiyama M (1996) Protective effect of vitamin E on chromium (VI)-induced cytotoxicity and lipid peroxidation in primary cultures of rat hepatocytes. *Arch Toxicol* **71**: 20–24

Verstraeten SV, Nogueira LV, Schreier S and Oteiza PI (1997) Effect of trivalent metal ions on phase separation and membrane lipid packing: role in lipid peroxidation. *Arch Biochem Biophys* **338**: 121–127

Wartenberg M, Hescheler J and Sauer H (1997) Electrical fields enhance growth of cancer spheroids by reactive oxygen species and intracellular $Ca^{2+}$. *Am J Physiol* **272**: R1677–R1683

Wartenberg M, Frey C, Diedershagen H, Ritgen J, Hescheler J and Sauer H (1998*a*) Development of an intrinsic P-glycoprotein-mediated doxorubicin resistance in quiescent cell layers of large, multicellular prostate tumor spheroids. *Int J Cancer* **75**: 855–863

Wartenberg M, Hescheler J, Acker H, Diedershagen H and Sauer H (1998*b*) Doxorubicin distribution in multicellular prostate cancer spheroids evaluated by confocal laser scanning microscopy and the ‘optical probe technique’. *Cytometry* **31**: 137–145

Weaver JC (1993) Electroporation: a general phenomenon for manipulating cells and tissues. *J Cell Biochem* **51**: 426–435

Wedekind P, Kubitschek U, Heinrich O and Peters R (1996) Line-scanning microphotolysis for diffraction-limited measurements of lateral diffusion. *Biophys J* **71**: 1621–1632

*© 1999 Cancer Research Campaign*
